# Persistent Poor Health after COVID-19 Is Not Associated with Respiratory Complications or Initial Disease Severity

**DOI:** 10.1513/AnnalsATS.202009-1175OC

**Published:** 2021-06-01

**Authors:** Liam Townsend, Joanne Dowds, Kate O’Brien, Grainne Sheill, Adam H. Dyer, Brendan O’Kelly, John P. Hynes, Aoife Mooney, Jean Dunne, Cliona Ni Cheallaigh, Cliona O’Farrelly, Nollaig M. Bourke, Niall Conlon, Ignacio Martin-Loeches, Colm Bergin, Parthiban Nadarajan, Ciaran Bannan

**Affiliations:** ^1^ Department of Infectious Diseases; ^3^ Department of Physiotherapy; ^7^ Department of Immunology; ^11^ Department of Intensive Care Medicine, and; ^12^ Department of Respiratory Medicine, St. James’s Hospital, Dublin, Ireland; ^2^ Department of Clinical Medicine and; ^5^ Department of Medical Gerontology, School of Medicine, Trinity Translational Medicine Institute; ^4^ Department of Physiotherapy; ^8^ School of Biochemistry and Immunology, Trinity Biomedical Sciences Institute, and; ^9^ Department of Comparative Immunology and; ^10^ Department of Immunology, School of Medicine, Trinity College, Dublin, Ireland; and; ^6^ Department of Radiology, Mater Misericordiae University Hospital, Dublin, Ireland

**Keywords:** COVID-19, respiratory complications, breathlessness

## Abstract

**Rationale:** Much is known about the acute infective process of severe acute respiratory syndrome coronavirus 2 (SARS-CoV-2), the causative virus of the coronavirus disease (COVID-19) pandemic. The marked inflammatory response and coagulopathic state in acute SARS-CoV-2 infection may promote pulmonary fibrosis. However, little is known about the incidence and seriousness of post–COVID-19 pulmonary pathology.

**Objectives:** To describe the respiratory recovery and self-reported health after infection at the time of outpatient attendance.

**Methods:** Infection severity was graded into three groups: *1*) not requiring admission, *2*) requiring hospital admission, and *3*) requiring intensive care unit care. Participants underwent chest radiography and a 6-minute walk test (6MWT). Fatigue and subjective return to health were assessed, and concentrations of CRP (C-reactive protein), IL-6 (interleukin-6), sCD25 (soluble CD25), and D-dimer were measured. The associations between initial illness and abnormal chest X-ray findings, 6MWT distance, and perception of maximal exertion were investigated.

**Results:** A total of 487 patients were offered an outpatient appointment, of whom 153 (31%) attended for assessment at a median of 75 days after diagnosis. A total of 74 (48%) had required hospital admission during acute infection. Persistently abnormal chest X-ray findings were seen in 4%. The median 6MWT distance covered was 460 m. A reduced distance covered was associated with frailty and length of inpatient stay. A total of 95 (62%) patients believed that they had not returned to full health, whereas 47% met the case definition for fatigue. Ongoing ill health and fatigue were associated with an increased perception of exertion. None of the measures of persistent respiratory disease were associated with initial disease severity.

**Conclusions:** This study highlights the rates of objective respiratory disease and subjective respiratory symptoms after COVID-19 and the complex multifactorial nature of post–COVID-19 ill health.

Coronavirus disease (COVID-19), caused by severe acute respiratory syndrome coronavirus 2 (SARS-CoV-2), has led to a global pandemic (1). The clinical and pathological features of acute infection have been extensively published, with a wide spectrum of disease seen, from asymptomatic infection to mild self-limiting symptoms to acute respiratory failure and the need for invasive mechanical ventilation (2, 3). A clinical picture similar to that of acute respiratory distress syndrome (ARDS) with refractory hypoxemia is the primary cause of death in COVID-19 (4). There is a paucity of data surrounding the potential consequences and sequelae of infection. ARDS is a fibroproliferative disease, with lung biopsy specimens taken at the time of ARDS showing fibrosis in more than half of affected patients (5, 6). The marked inflammatory response and coagulopathic state in response to SARS-CoV-2 may promote pulmonary fibrosis and lung damage (7–9). Looking at the follow-up of patients with SARS from 2003, there are a small number who developed postinfectious fibrosis, with less than 5% of admitted patients being affected (10, 11). There is an array of differing radiological appearances during acute COVID-19 (12). These changes are not limited to those seen in ARDS, and this has impacted ventilatory management strategies for severe COVID-19 (13–15). Thus COVID-19 pneumonitis may have lasting effects, even in the absence of ARDS (16).

Furthermore, acute SARS-CoV-2 infection is not limited to the respiratory system and has multisystem effects, with evidence of cardiovascular, coagulation, and gastrointestinal system disturbance in addition to the primary pulmonary disease (17–19). There has been recent interest in the possibility of so-called “long COVID-19,” whereby patients have persistence of a multitude of symptoms after initial infection resolution (20). The most common complaints reported after COVID-19 are breathlessness and persistent fatigue, although the mechanisms underlying this are unclear (21). However, breathlessness is subjective and multifactorial and may not be due to respiratory compromise (22).

We set out to evaluate medium-term respiratory complications after SARS-CoV-2 infection, as defined by chest radiography, distance covered, and maximal perceived exertion measured by using a 6-minute walk test (6MWT). This is in line with recently published algorithms for the respiratory follow-up of patients with COVID-19 (23). These algorithms recommend that all patients undergo chest radiography, with further testing suggested for those with abnormal imaging findings. We evaluated these assessments in the context of the severity of the initial infection as well as in the context of ongoing inflammation at follow-up. We investigated persistent ill health by also including subjective measures of fatigue and returning to health.

## Methods

### Study Setting and Participants

This cross-sectional study was performed in the post–COVID-19 review clinic at St. James’s Hospital, Dublin, Ireland. Informed written consent was obtained from all participants in the current study in accordance with the Declaration of Helsinki (24). Ethical approval for the current study was obtained from the Tallaght University Hospital/St. James Hospital Joint Research Ethics Committee (reference Research Ethics Committee 2020-04 [01]). Participants who had a positive SARS-CoV-2 polymerase chain reaction result at our institution in the 3-month period from March to May 2020 were recruited from the post–COVID-19 outpatient clinic. This included those managed as inpatients and staff members in whom COVID-19 was diagnosed at our center but who self-managed at home. Patients were not offered an outpatient appointment if they were residents in long-term care facilities. Patients attending the outpatient clinic were invited to participate in the current study by a research physician. To be considered for inclusion in the current study, participation had to occur at least 6 weeks after *1*) the date of last acute COVID-19 symptoms (for outpatients) and/or *2*) the date of discharge for those who were admitted during their acute COVID-19 illness.

### Clinical Covariate Assessment

All data were obtained at the time of outpatient assessment. Routine demographic information was collected from participants. Further information was obtained from patient records and included the dates of COVID-19 symptoms, inpatient admission, and admission to the intensive care unit (ICU). Peak oxygen requirements, peak CRP (C-reactive protein) concentrations and abnormal chest X-ray findings during acute infection were also recorded for those admitted. The chest X-rays were scored using the *Brixia* system by a radiologist blinded to the patient outcome. The *Brixia* score was proposed as an objective severity measure for chest X-ray abnormalities in acute COVID-19 (25). This score attributes a score of 0–3 for each lung zone on the basis of the severity of changes seen, with a maximum score of 18 indicating the most severe disease. It has been shown to be associated with disease severity and mortality (26, 27). Patients were divided into nonadmitted and admitted groups to allow for COVID-19 illness severity assessment. The admitted group was further subdivided into those who required ICU care and those who did not. Participants were assessed for frailty by a member of the COVID-19 clinical team, which was operationalized using Rockwood’s Clinical Frailty Scale (range, 1–9) (28). Patients underwent chest X-ray at the time of their outpatient appointment to assess for the presence of parenchymal disease and the resolution of any previously seen radiological abnormalities (29); CRP, IL-6 (interleukin-6), sCD25 (soluble CD25), and D-dimer were also measured in serum by using an enzyme-linked immunosorbent assay (R&D Systems).

A 6MWT was used to assess cardiopulmonary and musculoskeletal function (30, 31). It has been previously validated in the assessment of survivors of ARDS (32). The results of the 6MWT were compared with normative reference ranges for both a healthy population and a previously published ARDS population (32). Total distance walked and significant desaturation (defined as an oxygen saturation below 90%) were recorded. The Modified Borg Dyspnea Scale (MBS) was used to assess perceived exertion during submaximal exercise (range, 0–10). The MBS has been widely used in both healthy and diseased states (33, 34).

To assess subjective recovery from COVID-19 illness, participants were asked a binary question regarding their perception of having returned to full health. Fatigue was assessed using the validated Chalder Fatigue Scale (35, 36). Participants are asked to answer these questions with reference to the past month in comparison with their pre–COVID-19 baseline, with responses measured on a Likert scale (0–3). From this, a global score can be constructed out of a total of 33. This method is validated and closely resembles other fatigue questionnaires (37–40).

### Statistical Analysis

All analysis was performed using Stata version 15.0 (StataCorp). Statistical significance was indicated by *P* < 0.05.

We examined characteristics and outcomes according to acute COVID-19 severity: *1*) nonadmitted, *2*) admitted but not requiring ICU care, and *3*) admitted to the ICU. Analysis of variance, Kruskal-Wallis tests, and chi-square tests were used as appropriate for univariate between-group differences. Tukey or Dunn testing was used *post hoc*. Pearson and Spearman tests were used to assess the correlation between variables.

To analyze the impact of disease severity on respiratory outcomes, we used linear regression, with each outcome (namely 6MWT distance covered and maximal MBS) as the dependent variable. We additionally analyzed factors associated with abnormal chest X-ray findings at the time of study participation. This was done using binary logistic regression. Models were adjusted for the confounding variables of age, sex, and clinical frailty score. Results are reported as β coefficients or odds ratios (ORs) with 95% confidence intervals (CIs) and corresponding *P* values. Linear models were examined for multicollinearity by computing variance inflation factors and visually examining residual-versus-fit plots.

## Results

### Cohort Descriptors

**Figure 1.**
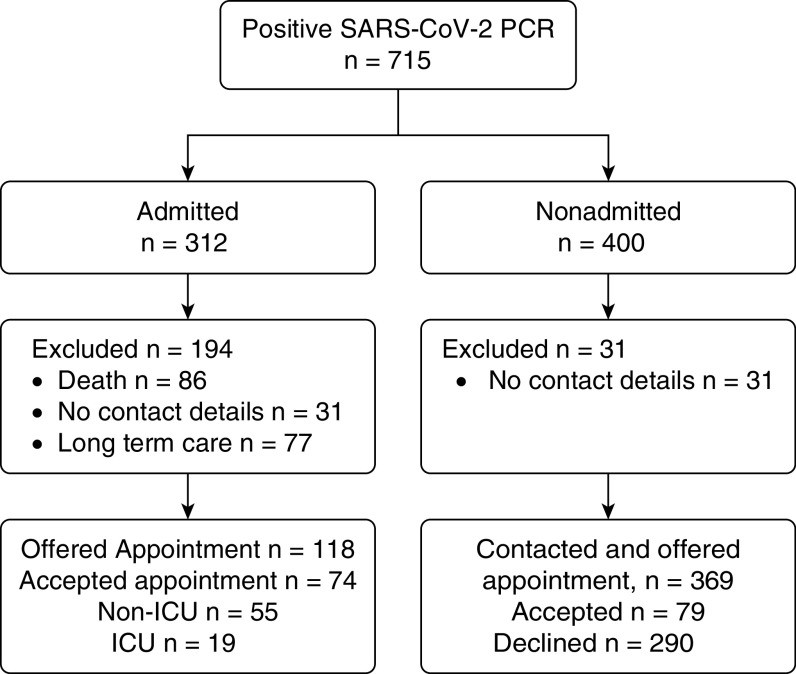
Patient enrollment diagram. ICU = intensive care unit; PCR = polymerase chain reaction; SARS-CoV-2 = severe acute respiratory syndrome coronavirus 2.

SARS-CoV-2 was diagnosed in 712 patients at our institution in the 3-month period from March to May 2020. A total of 400 (56%) were managed in the outpatient setting, with 312 (44%) admitted and 44 (44/312, 14%) requiring ICU care. Of the 712 patients, 86 (12%) died. After exclusion of those admitted patients who were long-term care residents or had no contact details, 118 were contacted and offered an appointment, of whom 74 (63%) attended. Of the 400 patients who self-managed in the community, 31 had no contact details. A total of 369 were contacted, with 79 (21%) accepting an outpatient appointment. Thus 153 patients of a contactable 487 (31%) attended for follow-up. Of these, just under half (74/153, 48%) had been admitted during their acute infection and 19 of 153 (12%) were admitted to an ICU. The enrolment strategy and breakdown are shown in Figure 1. The median age of the outpatient cohort was 48 years (range, 35–59 yr). The median time of follow-up was 75 days after diagnosis (interquartile range [IQR], 66–108 d). Those managed as inpatients had a shorter time to follow-up (median, 71 d; IQR, 61–82 d) than those managed in the community (median, 92 d; IQR, 62–117 d) (*P* < 0.001).

Characteristics of the cohort are given by disease severity category in Table 1. There were significant differences among the groups with respect to sex, age, and Clinical Frailty Score. The admitted group (both non-ICU and ICU admissions) had an increased proportion of males, were older, and had greater degrees of frailty than those in the nonadmitted group (all *P* < 0.001).

**Table 1.** Cohort demographics, outpatient results, and inpatient severity markers

Results at Time of Initial Infection	Nonadmitted (*N* = *79*)	Admitted, Non-ICU (*N* = *55*)	Admitted, ICU (*N* = *19*)
Peak CRP, mg/ml, mean (SD)	—	64.4 (70.3)	178.6 (102.6)
Peak Fi_O_2__, %, mean (SD)	—	30 (15)	77 (20)
Abnormal CXR, *n* (%)	—	32 (58)	19 (100)
*Brixia* score, mean (SD)	—	4.38 (3.37)	7.84 (4.69)
Length of stay, d, median (IQR)	—	7 (5–13)	22 (14–29)
Demographics and results at the time of outpatient assessment			
Age, yr, mean (SD)	40.2 (11.4)	56.4 (15.5)	54.5 (11.6)
Sex, female, *n* (%)	57 (72.2)	26 (47.3)	5 (26.3)
Ethnicity, *n* (%)			
White	56 (70.9)	45 (81.8)	14 (73.7)
Asian	17 (21.5)	6 (10.9)	3 (15.8)
Hispanic	2 (2.5)	0 (0)	0 (0)
African	4 (5.1)	4 (7.3)	2 (10.5)
Clinical Frailty Score, median (IQR)	1 (1–1)	2 (2–3)	2 (1–3)
Back to full health, *n* (%)	27 (34)	27 (59)	9 (47)
Fatigue score, median (IQR)	17 (12–21)	15 (11–21)	14 (11–15)
CRP, mg/ml, mean (SD)	2.12 (2.01)	3.61 (4.85)	1.60 (0.98)
IL-6, pg/ml, mean (SD)	3.55 (1.51)	4.58 (2.95)	3.54 (0.76)
sCD25, pg/ml, mean (SD)	825.47 (398.40)	1,503.31 (777.76)	1,534.18 (588.60)
D-dimer, ng/ml, mean (SD)	415.3 (775.5)	669.1 (799.9)	454.1 (209.9)

*Definition of abbreviations*: CRP = C reactive protein; CXR = chest X-ray; Fi_O_2__ = fraction of inspired oxygen; ICU = intensive care unit; IL-6 = interleukin-6; IQR = interquartile range; sCD25 = soluble CD25; SD = standard deviation.

There were no differences in demographics between those admitted but not requiring ICU care and those admitted to an ICU, but those admitted to an ICU had a longer inpatient stay.

### Respiratory Sequalae

A total of 115 (75%) participants underwent chest radiography at follow-up. All patients admitted during acute infection underwent a follow-up chest X-ray. Abnormal chest X-ray findings were found in 51 of 74 (69%) admitted patients at the time of initial infection. Persistent abnormal X-ray findings of either persistent infiltrate or atelectasis were found in 14 (19%) of the admitted cohort, with no abnormal findings in those managed as outpatients. Abnormal chest X-ray findings were not associated with initial disease severity. These results are summarized in Table 2.

**Table 2.** Relationship between respiratory outcomes and disease severity

	Abnormal Chest X-Ray		Distance at 6MWT		Maximal Borg Dyspnea Scale Score during 6MWT	
	OR (95% CI)	*P* Value	β Coefficient (95% CI)	*P* Value	β Coefficient (95% CI)	*P* Value
Disease severity						
Nonadmitted	1.0 (reference)	—	0 (reference)	—	0 (reference)	—
Admitted, non-ICU	2.6 (0.5 to 14.0)	0.26	−23.6 (−69.4 to 22.2)	0.31	−0.15 (−1.3 to 1.0)	0.79
Admitted, ICU	4.9 (0.8 to 30.1)	0.09	−27.1 (−85.2 to 31.0)	0.36	−0.56 (−2.0 to 0.9)	0.45
Age	1.0 (1.0 to 1.1)	0.39	−1.8 (−3.4 to −0.3)	0.02	0.01 (−0.03 to 0.04)	0.78
Sex, female	1.1 (0.3 to 3.9)	0.85	−47.1 (−83.4 to −10.7)	0.01	0.95 (0.04 to 1.9)	0.04
CFS	0.9 (0.4 to 2.1)	0.81	−47.4 (−73.0 to −21.8)	<0.001	0.65 (0.01 to 1.3)	0.048

*Definition of abbreviations*: 6MWT = 6-minute walk test; CFS = Clinical Frailty Score; CI = confidence interval; ICU = intensive care unit; OR = odds ratio.

Length of hospital stay was associated with an increased likelihood of an abnormal X-ray finding, although other markers of disease severity showed no association (*see* Table E1 in the online supplement).

All 14 patients who had an abnormal follow-up X-ray finding underwent repeat imaging 6 weeks later, with 5 having persistent abnormalities. Subsequent computed tomographic (CT) scans of these five patients demonstrated scarring in one lung zone (three patients) and two lung zones (two patients). Thus, persistent X-ray abnormalities attributable to COVID-19 were seen in 4% (5/115) of our cohort.

### 6MWT and MBS

A total of 109 (71%) patients completed a 6MWT. The median distance covered was 460 m (IQR, 225–640 m). The distance covered was not associated with initial disease severity. These results are shown in Table 2. The length of inpatient stay was associated with the reduced distance covered, but no other features of initial infection were associated with the distance covered (Table E1). Three patients (3/109, 3%) had an arterial oxygen saturation below 90% during the 6MWT. The median MBS score reported was 3 (IQR, 2–5). Maximal scores of perceived exertion were independent of initial disease severity (Table 2). The only predictor of an increased MBS score in the inpatient cohort was female sex (Table E1).

### Fatigue and Ill Health

A total of 95 (62%) patients did not feel back to full health at the time of their outpatient assessment. The median fatigue score across the cohort was 15 (IQR, 11–20). A total of 73 (48%) participants met the case definition for fatigue, and this was not associated with the severity of initial infection (*r* ^2^ = −0.09; *P* = 0.25). Not feeling back to full health was associated with an increased MBS score (OR, 1.3; 95% CI, 1.1–1.6; *P* = 0.005). Similarly, fatigue was not associated with abnormal chest X-ray findings or inflammatory markers at follow-up but was associated with reduced distance covered (β coefficient = −0.02; 95% CI, −0.03 to −0.01; *P* = 0.002) and an increased MBS score (β coefficient = 0.85; 95% CI, 0.36–1.334; *P* = 0.001). Fatigue and failure to feel back to full health were also closely correlated (β coefficient, 5.34; 95% CI, 3.64 to 7.05; *P* < 0.001).

## Discussion

Of the patients who attended for outpatient follow-up, we report reassuring findings regarding objective post–COVID-19 respiratory complications at a median follow-up time point of 75 days but report clear evidence that patients have not returned to full fitness. Our pragmatic approach of repeat radiography followed by CT scans for persistent abnormalities produced an abnormal imaging rate of 4%. There was also no association with abnormal imaging and severe disease. The 6MWT distance covered by our cohort is below that reported in the healthy population (41). This is similar to data seen after the original SARS pandemic in 2003 (42). However, the distance covered in our cohort is higher than those previously reported at 3-month and 6-month follow-ups of patients with ARDS (32). The median distance covered is also higher than 350 m, which has previously been shown to be associated with hospital admission and all-cause mortality in patients with preexisting pulmonary disease (43). Under multivariate analysis, no disease-specific characteristics affected the distance covered. The presence of normal chest radiographic findings in the majority of our cohort, coupled with very low rates of significant desaturation during the 6MWT (3%) and a relatively large cohort size, suggest that clinically relevant fibrosis is an uncommon consequence of SARS-CoV-2 infection. This combination of readily available tests helps overcome the relative lack of sensitivity of chest X-rays in the diagnosis of pulmonary fibrosis (44).

There are scant data available from other sites with which to compare our radiological findings. A single study in China performed high-resolution CT scans on 55 patients at 3 months, finding abnormalities in 39 of these, but symptomatic dyspnea was reported in fewer than 15%, and three-quarters had normal pulmonary function test results (45). Fibrosis has been shown at biopsy in approximately half of the patients with all-cause ARDS at the time of acute illness (6). However, ARDS-associated fibrosis is also closely linked with mortality, so it is plausible that those with significant fibrosis did not survive to follow-up (46).

Significant morbidity persists, with 62% of patients reporting that they have not returned to full health. Fatigue is a common complaint in our cohort, confirming early reports from other centers (47). This failure to return to full health and presence of deconditioning is supported by the associations among fatigue, subjective perception of not returning to full health, and increased perception of maximal exertion. In addition to deconditioning, persistent low-grade inflammation after infection may also contribute to systemic ill health (48). These results support the need for more in-depth cardiovascular health and fitness assessment of those most severely affected, including cardiac imaging and maximal-oxygen-uptake assessment (49, 50).

Our findings are reflective of the 2003 SARS outbreak, with survivors reporting impairments in health-related quality of life at 6 months (*n* = 110) (42). Similarly, a subset of patients in Toronto experienced persistent fatigue, diffuse myalgia, weakness, and depression 1 year after their acute illness and could not return to work (51). Over 40% of 233 survivors of SARS in Hong Kong reported a chronic fatigue problem 40 months after infection (52).

Our study concerns findings around COVID-19–related sequelae in the medium term. The median follow-up was more than 10 weeks after diagnosis, and participants were deemed recovered from initial infection, in line with what is currently understood of viral dynamics and infectivity (53–55). We believe that the findings are noteworthy on two main fronts. First, they are reassuring regarding the long-term respiratory impact of COVID-19. Second, we have demonstrated the significant morbidity that persists after infection, affecting the perception of health, the ability to return to work, and the presence of enduring fatigue. This morbidity appears to be unrelated to initial infection severity. This has implications for both the delivery of adequate health care to all patients with diagnosed COVID-19, irrespective of the need for hospitalization, as well as the economic impact on the workforce. There appears to be a need for ongoing support and rehabilitation of patients experiencing long-term side effects of COVID-19, including programs to optimize patients’ self-management of fatigue and perception of exertion after COVID-19 (56). This is a topic that has been garnering much attention, but there is very limited published evidence thus far (57). We hope that this study can help inform ongoing decisions regarding the management of post–COVID-19 symptoms.

### Limitations

Our single-center study has several limitations worth noting. We were unable to assess the proportion of patients who declined an outpatient appointment. Telephone follow-up was not feasible because of resource constraints. It is possible that those who have persistent ill health are overrepresented in our cohort. Similarly, we cannot generalize our objective respiratory results to the total COVID-19 cohort. We report on a predominantly white population, which may not be generalizable. It is a cross-sectional study at a single time point. Therefore, we suggest an ongoing assessment of those displaying persistent ill health. Furthermore, participants did not undergo a 6MWT before infection, so changes from baseline are difficult to assess.

### Conclusions

We present the medium-term respiratory and self-perceived health of patients at a median of 75 days after diagnosis. We found little evidence for postinfectious pulmonary fibrosis on chest X-rays or for hypoxia during 6MWTs. However, 62% of patients did not feel back to full health, and this was associated with an increased perception of exertion. A total of 47% of our cohort met the diagnostic criteria for fatigue, independent of the initial severity of the infection. This study highlights the persistence of ill health after SARS-CoV-2 infection that presents a serious burden to quality of life. The lack of association with infection severity highlights that this may be an issue for a large number of patients, and this should be used to inform management strategies for convalescent patients.
